# Efficiency and determinants of science and technology investment in Chinese competitive sports: a provincial DEA-Tobit analysis

**DOI:** 10.3389/fspor.2026.1738361

**Published:** 2026-01-26

**Authors:** Rui Xia, Shihan Lu, Lunan Zhao

**Affiliations:** 1 College of Sports Science, Qufu Normal University, Qufu, China; 2 School of Physical Education and Sports Science, South China Normal University, Guangzhou, China

**Keywords:** China, competitive sports, DEA-Tobit model, resource-based view, science and technology investment

## Abstract

**Introduction:**

The efficiency with which science and technology (S&T) investment is translated into competitive sports performance is a critical yet underexplored issue in China, despite the rapid growth of related funding in recent years.

**Methods:**

This study evaluates the efficiency of provincial S&T investment in competitive sports across 31 Chinese provinces from 2018 to 2022. Using a two-stage analytical framework grounded in the Resource-Based View, we integrate Data Envelopment Analysis (DEA), the Malmquist productivity index, and Tobit regression to assess static efficiency, dynamic productivity changes, and their key influencing factors.

**Results:**

The results indicate that the overall efficiency of S&T investment in China's competitive sports system remains relatively low, with pronounced regional disparities. Dynamic analysis reveals that total factor productivity declined on average, primarily due to limited technological progress rather than changes in technical efficiency. Tobit regression results show that research staff input and the emphasis on technological R&D are negatively associated with efficiency, while regional economic development improves pure technical efficiency but undermines scale efficiency.

**Conclusion:**

These findings suggest that the main constraint on the effectiveness of S&T investment lies not in the quantity of resources but in their strategic configuration and conversion into athletic outcomes. Policy efforts should therefore shift from expanding investment scale toward optimizing resource orchestration and strengthening the linkage between scientific research and training practice to enhance the overall efficiency and sustainability of competitive sports development in China.

## 1 Introduction

Competitive sports function not only as a reflection of national culture and strength but also as an important indicator of economic and social development (1, 2). Over recent decades, China has achieved remarkable success in major international sporting competitions. These achievements have been strongly supported by sustained investment in science and technology (S&T). From 2018 to 2022, China's S&T investment in competitive sports increased by nearly 50%, reflecting strong national prioritization and an increasing reliance on technological advancement. However, the efficiency of this substantial investment—how effectively resources are converted into sporting success—remains a critical question that warrants in-depth exploration. The effectiveness of S&T input therefore depends critically on rational resource allocation and efficient utilization.

To evaluate such efficiency, Data Envelopment Analysis (DEA) has been widely employed in sports research because it accommodates multiple inputs and outputs without requiring a pre-specified production function (3–5). However, standard DEA models are limited in their ability to account for stochastic disturbances and endogeneity when examining the determinants of efficiency. The Tobit model, which is well suited for truncated dependent variables such as efficiency scores, is therefore commonly used to complement DEA analysis (6, 7). Integrating the DEA and Tobit models leverages the strengths of both, allowing not only for an accurate assessment of S&T investment efficiency but also for a deeper exploration of the factors that influence it.

Existing research on S&T investment in sports has primarily focused on macro-level benefit analyses or on the application of specific technologies in isolated domains (8–11). There is a lack of systematic studies evaluating the overall efficiency of S&T investment and its influencing factors, particularly from a comprehensive, provincial-level perspective in China. Moreover, the determinants of efficiency are complex and multidimensional—encompassing policy, human resources, and organizational management—yet the mechanisms through which these factors operate remain insufficiently explored.

To ground this empirical investigation theoretically, the study adopts the Resource-Based View (RBV) as its overarching framework. It conceptualizes the S&T inputs—funding, institutions, and personnel—as strategic resources for competitive sports development. Accordingly, the core research question shifts from “what is the level of efficiency?” to “how effectively do different regions convert strategic S&T resources into competitive performance?”. This perspective inherently directs attention to the conversion mechanisms and contextual factors that explain efficiency disparities, which the subsequent DEA and Tobit models are designed to measure and analyze.

In light of these considerations, this study employs the DEA-Tobit model to systematically evaluate the efficiency of S&T investment in China's competitive sports development across 31 provinces from 2018 to 2022 and to analyze the key factors influencing this efficiency. The aim is to provide theoretical support and practical guidance for optimizing S&T resource allocation and enhancing the quality of competitive sports development.

## 2 Literature review

### 2.1 The application of the DEA-Tobit model in the field of sports

The DEA–Tobit model, which combines Data Envelopment Analysis (DEA) for efficiency measurement with Tobit regression for determinant analysis, has become a widely adopted approach in studies of sports efficiency. DEA has been extensively used to assess the efficiency of public sports services, the sustainable development of competitive sports, and the integration of the sports industry with tourism (12–17). For example, prior studies have applied DEA to measure provincial efficiency in public sports services by incorporating environmental variables such as GDP, and to analyze development trends in competitive sports (12, 13). Tobit regression is then employed to examine the effects of environmental and managerial factors on DEA-derived efficiency scores, thereby addressing DEA's limitations in handling exogenous influences. Collectively, these applications demonstrate the model's utility in diagnosing efficiency and guiding resource optimization. However, existing research has predominantly focused on general sports services or industrial efficiency, while systematic and longitudinal analyses specifically examining the efficiency of S&T investment in competitive sports—and its determinants across diverse regional contexts—remain scarce.

### 2.2 The impact of scientific and technological input on the development of competitive sports

Science and technology (S&T) input is widely recognized as a critical driver of modern competitive sports, underpinning improvements in training effectiveness, competitive performance, and decision-making processes. Technological tools—including biomechanical analysis, physiological monitoring, smart wearable devices, and data mining techniques—play an increasingly important role in informing athlete training and tactical planning (15, 16). For instance, in table tennis, data mining systems enable fine-grained technical and tactical analysis, whereas in track and field, advanced sensor technologies enhance the accuracy of motion capture (15, 16). Furthermore, applications in sports medicine and virtual reality (VR) training support athlete health management and the development of real-time decision-making capabilities (17, 18). These cases underscore that S&T investment permeates all aspects of competitive sports, from talent cultivation to elite performance. However, much of the existing literature examines the effects of specific technologies in isolation. A comprehensive, system-level evaluation of the efficiency of bundled S&T resources—including funding, institutions, and personnel—remains underexplored.

### 2.3 Existing studies on the influential factors of S&T investment

Prior studies on the determinants of S&T investment efficiency emphasize the roles of the policy environment, human resources, and research and development (R&D) systems. Government policy support and fiscal investment are widely regarded as important drivers of technological development and resource allocation efficiency in the sports sector (19–21). In addition, the efficiency of public R&D investment and the structure of fiscal decentralization have been shown to influence technological innovation and its diffusion and application at the local level (22, 23). Nevertheless, existing studies have paid limited attention to the mechanisms through which multidimensional factors—such as regional economic development, management input, and the alignment between research activities and sports practice—interact to shape the efficiency with which S&T resources are converted into athletic outcomes. This gap highlights the need for an integrated analytical framework that simultaneously evaluates efficiency and examines the influence of contextual factors—an objective addressed in the present study.

## 3 Materials and methods

### 3.1 Materials

#### 3.1.1 Evaluation index selection

Guided by the Resource-Based View (RBV), this study conceptualizes science and technology (S&T) input as a systematic configuration of financial, human, and institutional resources dedicated to enhancing athletic training, performance, and competitive outcomes. Consistent with RBV principles, the empirical analysis follows a two-stage logic. First, the Data Envelopment Analysis (DEA) model is employed to assess the relative efficiency with which bundled S&T resources are transformed into competitive sports achievements, thereby capturing regional capabilities in resource conversion. Second, a Tobit regression model is used to examine how contextual and environmental factors influence this conversion process and to explain observed efficiency differentials across regions.

Based on this analytical framework, an input–output indicator system was constructed. The input indicators reflect four key dimensions of S&T resources: financial investment in scientific research (X1), institutional infrastructure (X2), fiscal support for S&T research and development (X3), and human resource input measured by the number of research personnel (X4). The output indicators correspond to the core objectives of competitive sports development, including the production of elite athletes (Y1), the sustainability and quality of the talent pipeline as proxied by the size of the referee team (Y2), and competitive performance measured by the number of world champions (Y3). Detailed definitions and measurement units of all indicators are provided in Table 1.

**Table 1:** Evaluation indicators.

Category	Indicator	Specific indicator	Definition	Unit
Input	Financial investment in scientific research	Investment of science and technology funds in various provinces and cities (X1)	Total funds for scientific research projects in each province and city within one year	Ten thousand yuan
	Material input	Number of sports science research structures in provinces and cities (X2)	Number of scientific research institutions in this province as of the statistical year.	Number of people
	Investment in scientific and technological research and development	Financial revenue support of science and technology amount by province (X3)	The annual financial allocation of each province is used for scientific and technological research and development.	Ten thousand yuan
	Human resource input	Number of employees in sports scientific research institutions in provinces and cities (X4)	The total number of employees in scientific research institutions in each province each year, including managers and professional and technical personnel.	Number of people
Output	Number of professional athletes	Number of elite athletes by province (Y1).	The number of athletes who have been certified as masters by the China Athletes’ Grade.	Individuals
	Size of referee team	Number of national referees and above in each province (Y2).	Including national and international referees.	Individuals
	Prize record	Each province won the number of world champions that year (Y3).	The number of world champions won on behalf of the province or institutions (such as universities) in the province.	Number of people

The empirical data were obtained from the Statistical Yearbook of Sports Affairs and official publications of the General Administration of Sport of China, covering 31 provinces from 2018 to 2022. These provinces are treated as decision-making units (DMUs) in the efficiency analysis. With respect to the output indicators, the number of elite athletes and world champions was selected as representative measures of competitive sports performance. Although these outcomes may exhibit a time-lag effect—reflecting the cumulative impact of long-term investment rather than immediate technological input—they remain the most standardized and authoritative indicators within China's provincial sports evaluation system. Moreover, in contemporary competitive sports, technological support is increasingly embedded in the daily training, monitoring, and performance optimization of existing elite athletes. Accordingly, these indicators are considered a valid, albeit lagged, reflection of the effectiveness of the current S&T support system.

#### 3.1.2 Data sources

The data utilized in this study are derived from the China Statistical Yearbook and data compiled by the General Administration of Sport of China. The dataset spans the period from 2018 to 2022 and encompasses 31 provinces and municipalities in China. To ensure data integrity and the reliability of interprovincial comparisons, particular attention was paid to the treatment of missing values. Given that minimum-value substitution may bias efficiency estimates in DEA by artificially distorting the production frontier, a more robust imputation strategy was adopted. Specifically, missing observations for certain provinces were estimated using regional mean values or linear interpolation based on adjacent years, depending on data availability and temporal continuity. This approach helps prevent the introduction of extreme or implausible values, thereby preserving the stability of the DEA frontier and providing a more accurate representation of relative efficiency across provinces.

### 3.2 Methods

#### 3.2.1 DEA-BCC model

Data Envelopment Analysis (DEA) is a non-parametric method widely used to evaluate the relative efficiency of decision-making units (DMUs) with multiple inputs and outputs. Originally proposed by Charnes, Cooper, and Rhodes (CCR) (3) in the late 1970s, the classical DEA model assumes constant returns to scale. To relax this assumption, Banker, Charnes, and Cooper (24) subsequently developed the BCC model, which allows for variable returns to scale and decomposes overall technical efficiency into pure technical efficiency and scale efficiency.

In DEA analysis, inputs and outputs are incorporated into a linear programming framework to construct an empirical production frontier. An input-oriented specification is adopted when the objective is to minimize input usage while maintaining a given level of output. This orientation is particularly appropriate in contexts where output levels are largely constrained, and efficiency improvements are expected to arise from better resource utilization. As a non-parametric approach, DEA does not require prior specification of a production function or assumptions about the functional form, enabling an objective evaluation of efficiency across DMUs with heterogeneous input–output structures.


minθ

$$s\;t\;\overset{n}{\underset{i=1}{\sum }}\;\lambda_{i}X_{i}+S^{-}=\theta X_{i}$$

$$\overset{n}{\underset{i=1}{\sum }}\;\lambda_{i}Y_{i}+S^{+}\;=Y_{i}$$

$$\overset{n}{\underset{i=1}{\sum }}\;\lambda_{i}=1$$

$$\lambda_{i},\;S^{-},\;S^{+}\geq 0$$

Within the BCC framework, efficiency outcomes are reported in terms of technical efficiency (TE), pure technical efficiency (PTE), and scale efficiency (SE), with the relationship expressed as TE = PTE×SE. Pure technical efficiency reflects managerial and technological capability under a given scale of operations, whereas scale efficiency captures the extent to which a DMU operates at an optimal scale. A DMU is considered fully efficient only when TE equals 1, which occurs if and only if both PTE and SE equal 1. In the context of this study, a TE score of 1 indicates that a province efficiently converts its science and technology inputs into competitive sports outcomes under the prevailing technological and scale conditions.

#### 3.2.2 Malmquist index model

The Malmquist index is a widely used productivity measure for evaluating changes in efficiency and technology over time. Based on Data Envelopment Analysis (DEA) (7, 25), it enables the assessment of intertemporal variations in the performance of decision-making units (DMUs) without requiring a predefined production function. In this study, the Malmquist index is employed to examine dynamic changes in the efficiency of S&T investment in China's competitive sports system across different periods.

Following the decomposition proposed by Färe et al. (26), the Malmquist total factor productivity (TFP) index is expressed as the product of technical efficiency change (EC) and technological change (TC). Technical efficiency change reflects movements of a DMU toward or away from the production frontier under existing technology, whereas technological change captures shifts in the frontier itself, indicating technological progress or regress. When returns to scale are variable, EC can be further decomposed into pure technical efficiency change (PTEC) and scale efficiency change (SEC), allowing for a more nuanced interpretation of productivity dynamics. The formula for the Malmquist model (27) is as follows:

$$\mathrm{TFP}=M(y_{t\;=\;1},x_{t\;=\;1})=\frac{d^{t+1}(x_{t+\cdot 1,}y_{t+1})}{d^{t}(x_{t},y_{t})}{\left[\frac{d^{t}(x_{t+\cdot 1,}y_{t+1})}{d^{t+1}(x_{t+\cdot 1,}y_{t+1})}*\frac{d^{t}(x_{t},y_{t})}{d^{t+1}(x_{t},y^{t})}\right]}^{\frac{1}{2}}$$

In general, a Malmquist index value greater than 1 indicates an improvement in total factor productivity, a value equal to 1 denotes no change, and a value less than 1 indicates a decline in productivity. Similarly, values of EC or TC greater than 1 signify efficiency improvement or technological progress, respectively. By applying this model, the present study identifies the relative contributions of efficiency change and technological change to the evolution of S&T investment performance in competitive sports, thereby providing insight into whether productivity dynamics are driven primarily by managerial improvements or by advances in technology.

#### 3.2.3 Tobit model

In the second stage of the analysis, a Tobit regression model is employed to examine the determinants of efficiency in S&T investment across provinces. The use of the Tobit model is appropriate because the efficiency scores obtained from the DEA analysis are censored, taking values within the closed interval [0,1] (28). Conventional linear regression methods may therefore yield biased and inconsistent estimates.

Following standard practice, the DEA efficiency scores are treated as the dependent variable, while a set of explanatory variables capturing economic, institutional, and managerial conditions are included as independent variables. The Tobit model is estimated using maximum likelihood methods, allowing for consistent inference on the marginal effects of the explanatory factors on efficiency outcomes. The Tobit model is as follows:

$$y_{i}=\beta_{0}+\beta_{1}x_{1i}+\beta_{2}x_{2i}+\beta_{3}x_{3i}+\beta_{4}x_{4i}+\beta_{5}x_{5i}+\varepsilon_{i}$$

Through this framework, the model identifies how variations in regional economic development, management input, and research resource allocation are associated with differences in S&T investment efficiency. The estimated coefficients thus provide empirical evidence on the mechanisms through which contextual factors influence the effectiveness with which S&T resources are transformed into competitive sports performance.

## 4 Results and discussion

### 4.1 Static efficiency analysis

#### 4.1.1 Technical efficiency (TE) analysis

From 2018 to 2022, the average technical efficiency (TE) of S&T investment in competitive sports across China's provinces was 0.40, reflecting an overall low level of efficiency with significant regional disparities (Table 2).

**Table 2:** Analysis results of technical efficiency (TE) of S&T investment in provinces and cities.

DMU	2018	2019	2020	2021	2022
Beijing	0.007	0.278	0.116	<0.001	0.019
Tianjin	0.028	1.000	1.000	0.759	1.000
Hebei	0.022	0.095	0.040	0.003	0.007
Shanxi	0.004	0.224	0.086	<0.001	0.025
Inner Mongolia	1.000	0.427	1.000	0.833	1.000
Liaoning	0.020	1.000	1.000	1.000	1.000
Jilin	1.000	0.284	1.000	<0.001	0.027
Heilongjiang	0.018	0.405	0.088	<0.001	0.044
Shanghai	0.013	0.305	1.000	0.002	0.007
Jiangsu	0.004	0.111	0.033	<0.001	0.007
Zhejiang	0.030	1.000	0.492	0.001	0.028
Anhui	0.024	0.817	0.172	0.002	0.048
Fujian	0.014	0.705	0.158	0.001	0.022
Jiangxi	1.000	0.825	1.000	1.000	1.000
Shandong	0.012	0.373	1.000	0.001	0.082
Henan	1.000	1.000	1.000	1.000	1.000
Hubei	0.015	0.127	0.023	0.003	0.011
Hunan	0.005	0.383	0.137	<0.001	0.043
Guangdong	0.023	0.381	0.799	0.003	0.040
Guangxi	1.000	0.730	1.000	0.002	0.080
Hainan	1.000	1.000	1.000	0.180	0.217
Chongqing	0.010	0.625	1.000	0.001	1.000
Sichuan	0.035	1.000	1.000	0.003	0.016
Guizhou	0.006	0.353	0.032	<0.001	0.007
Yunnan	0.009	0.478	1.000	<0.001	1.000
Tibet	0.314	0.118	1.000	0.089	1.000
Shaanxi	0.014	0.194	1.000	<0.001	0.024
Gansu	0.008	0.715	0.249	<0.001	1.000
Qinghai	1.000	0.107	1.000	0.311	0.502
Ningxia	0.812	1.000	1.000	0.469	1.000
Xinjiang	0.006	0.689	0.585	0.002	0.069

Provinces such as Inner Mongolia, Liaoning, Henan, Jiangxi, Guangxi, and Qinghai consistently achieved high efficiency scores, often reaching the frontier value of 1.000 in most years. In contrast, provinces including Beijing, Hebei, Shanxi, Jiangsu, Hubei, Hunan, Guizhou, Tibet, Gansu, and Ningxia showed marked fluctuations and notably low efficiency scores in certain years—for instance, recording values near or equal to 0.000 in 2021 for several of these regions.

#### 4.1.2 Analysis of pure technical efficiency

An analysis of pure technical efficiency (PTE), which reflects managerial and technical capabilities under existing resource conditions, reveals that many provinces achieved optimal pure technical efficiency (PTE) (PTE = 1.000) in at least one year during the period (Table 3).

**Table 3:** Analysis results of pure technical efficiency of S&T investment in provinces and cities.

DMU	2018	2019	2020	2021	2022
Beijing	1.000	0.848	0.554	0.931	0.846
Tianjin	1.000	1.000	1.000	0.759	1.000
Hebei	0.743	0.614	0.562	1.000	0.486
Shanxi	0.437	0.313	0.207	0.567	0.572
Inner Mongolia	1.000	0.546	1.000	0.833	1.000
Liaoning	1.000	1.000	1.000	1.000	1.000
Jilin	1.000	0.878	1.000	0.465	0.434
Heilongjiang	0.858	0.748	0.622	0.612	0.805
Shanghai	1.000	1.000	1.000	1.000	0.925
Jiangsu	0.647	0.899	0.696	1.000	0.984
Zhejiang	1.000	1.000	1.000	1.000	0.909
Anhui	1.000	0.826	0.584	0.969	1.000
Fujian	0.961	1.000	0.726	1.000	0.642
Jiangxi	1.000	0.931	1.000	1.000	1.000
Shandong	1.000	1.000	1.000	1.000	1.000
Henan	1.000	1.000	1.000	1.000	1.000
Hubei	1.000	0.688	0.850	1.000	0.690
Hunan	0.701	0.547	0.301	0.957	0.647
Guangdong	1.000	1.000	1.000	1.000	1.000
Guangxi	1.000	0.750	1.000	1.000	1.000
Hainan	1.000	1.000	1.000	0.180	0.217
Chongqing	1.000	0.635	1.000	0.609	1.000
Sichuan	1.000	1.000	1.000	1.000	1.000
Guizhou	0.375	0.353	0.190	0.238	0.168
Yunnan	0.557	0.486	1.000	0.876	1.000
Tibet	0.314	0.118	1.000	0.089	1.000
Shaanxi	0.524	0.987	1.000	0.941	0.491
Gansu	0.364	0.715	0.420	0.787	1.000
Qinghai	1.000	0.107	1.000	0.311	0.502
Ningxia	0.812	1.000	1.000	0.469	1.000
Xinjiang	0.602	0.823	0.650	0.855	0.960

Provinces such as Tianjin, Liaoning, Shandong, Henan, Guangdong, and Sichuan sustained high PTE scores over multiple years. Notable fluctuations were evident in provinces including Hebei, Shanxi, Hunan, Guizhou, Yunnan, and Tibet. In contrast, Guizhou and Tibet consistently recorded lower PTE scores.

#### 4.1.3 Analysis of scale efficiency and Status

Scale efficiency scores indicated the appropriateness of the scale of operations (Table 4). Provinces including Tianjin, Inner Mongolia, Liaoning, Henan, Jiangxi, Hainan, Qinghai, and Ningxia exhibited constant returns to scale (SE = 1.000) in most years.

**Table 4:** Analysis of scale efficiency and status of S&T investment in provinces and cities.

DMU	2018	2019	2020	2021	2022
Beijing	0.007drs	0.328drs	0.210	<0.001	0.022drs
Tianjin	0.028drs	1.000-	1.000-	1.000-	1.00-
Hebei	0.03drs	0.155drs	0.071drs	0.003drs	0.014drs
Shanxi	0.009drs	0.717drs	0.417drs	0.001drs	0.043drs
Inner Mongolia	1.000-	0.782drs	1.000-	1.000-	1.000-
Liaoning	0.02drs	1.000-	1.000-	1.000-	1.000-
Jilin	1.000-	0.323drs	1.000-	0.001drs	0.063drs
Heilongjiang	0.021drs	0.541drs	0.141drs	0.001drs	0.055drs
Shanghai	0.013drs	0.305drs	1.000-	0.002drs	0.008drs
Jiangsu	0.006drs	0.123drs	0.048drs	<0.001	0.007drs
Zhejiang	0.03drs	1.000-	0.492drs	0.001drs	0.031drs
Anhui	0.024drs	0.989drs	0.294drs	0.002drs	0.048drs
Fujian	0.015drs	0.705drs	0.217drs	0.001drs	0.034drs
Jiangxi	1.000-	0.886drs	1.000-	1.000-	1.000-
Shandong	0.012drs	0.373drs	1.000-	0.001drs	0.082drs
Henan	1.000-	1.000-	1.000-	1.000-	1.000-
Hubei	0.0150drs	0.185drs	0.027drs	0.003drs	0.016drs
Hunan	0.007drs	0.7000drs	0.455drs	0.001drs	0.066drs
Guangdong	0.023drs	0.381drs	0.799drs	0.003drs	0.040drs
Guangxi	1.000-	0.973drs	1.000-	0.002drs	0.080drs
Hainan	1.000-	1.000-	1.000-	1.000-	1.000-
Chongqing	0.010drs	0.984drs	1.000-	0.002drs	1.000-
Sichuan	0.035drs	1.000-	1.000-	0.003drs	0.016drs
Guizhou	0.016drs	1.000-	0.166drs	0.001drs	0.040drs
Yunnan	0.017drs	0.983drs	1.000-	0.001drs	1.000-
Tibet	1.000-	1.000-	1.000-	1.000-	1.000-
Shaanxi	0.026drs	0.197drs	1.000-	0.001drs	0.048drs
Gansu	0.022drs	1.000-	0.593drs	0.001drs	1.000-
Qinghai	1.000-	1.000-	1.000-	1.000-	1.000-
Ningxia	1.000-	1.000-	1.000-	1.000-	1.000-
Xinjiang	0.010drs	0.837drs	0.900drs	0.002drs	0.072drs

“drs” denotes decreasing returns to scale, indicating that the decision-making unit (DMU) is operating at a scale where additional inputs yield proportionally smaller increases in output; “–” denotes constant returns to scale, indicating that the DMU is operating at the most productive scale size (MPSS), where scale efficiency is equal to 1.000.

Provinces such as Hebei, Shanxi, Jiangsu, Hubei, Hunan, Guizhou, Yunnan, Shaanxi, and Gansu showed lower or more volatile scale efficiency scores, often with values significantly below 1.000.

### 4.2 Dynamic efficiency analysis

#### 4.2.1 Overall analysis

The average Total Factor Productivity Change (TFPCH) over the period was 0.914, indicating an overall decline of 8.6% in dynamic efficiency (Table 5). Technical Efficiency Change (EFFCH) showed significant increases in Year 2 (10.128) and Year 5 (18.556), but a sharp drop in Year 4 (0.013). Technological Change (TECHCH) had a mean value of 0.748 (<1), suggesting limited contribution from frontier shifts. Pure Technical Efficiency Change (PECH) was relatively stable (mean = 0.988), while Scale Efficiency Change (SECH) showed improvement (mean = 1.237).

**Table 5:** Malmquist index summary of annual means.

Year	EFFCH	TECHCH	PECH	SECH	TFPCH
2	10.1280	0.0740	0.8680	11.6740	0.7470
3	0.9150	0.1870	1.0960	0.8340	0.1710
4	0.0130	22.4700	0.9300	0.0140	2.9210
5	18.5560	0.1010	1.0780	17.2190	1.8750
Mean	1.2230	0.7480	0.9880	1.2370	0.9140

#### 4.2.2 Provincial decomposition

Considerable variation existed across provinces (Table 6). Provinces like Tianjin (TFPCH = 3.893), Yunnan (1.616), Liaoning (1.466), and Chongqing (1.483) exhibited TFP growth (TFPCH >1). Others, such as Jilin (0.133), Guangxi (0.344), and Qinghai (0.291), experienced productivity decline. The mean TECHCH of 0.748 confirms the limited role of technological progress at the aggregate level.

**Table 6:** Malmquist index summary of firm means.

Firm	EFFCH	TECHCH	PECH	SECH	TFPCH
Beijing	1.267	0.719	0.959	1.322	0.911
Tianjin	2.454	1.586	1.000	2.454	3.893
Hebei	0.742	0.965	0.899	0.825	0.716
Shanxi	1.582	0.644	1.070	1.479	1.018
Inner Mongolia	1.000	0.795	1.000	1.000	0.795
Liaoning	2.648	0.554	1.000	2.648	1.466
Jilin	0.407	0.327	0.812	0.502	0.133
Heilongjiang	1.257	1.139	0.984	1.277	1.432
Shanghai	0.873	1.377	0.981	0.890	1.202
Jiangsu	1.149	0.843	1.110	1.034	0.968
Zhejiang	0.983	1.039	0.976	1.007	1.021
Anhui	1.196	0.574	1.000	1.196	0.686
Fujian	1.113	0.680	0.904	1.231	0.757
Jiangxi	1.000	0.923	1.000	1.000	0.923
Shandong	1.632	0.753	1.000	1.632	1.230
Henan	1.000	1.101	1.000	1.000	1.101
Hubei	0.939	1.010	0.911	1.030	0.948
Hunan	1.695	0.627	0.980	1.729	1.063
Guangdong	1.141	1.075	1.000	1.141	1.227
Guangxi	0.532	0.646	1.000	0.532	0.344
Hainan	0.682	0.752	0.682	1.000	0.513
Chongqing	3.134	0.473	1.000	3.134	1.483
Sichuan	0.826	1.277	1.000	0.826	1.054
Guizhou	1.036	0.461	0.818	1.267	0.477
Yunnan	3.199	0.505	1.158	2.763	1.616
Tibet	1.336	1.024	1.336	1.000	1.368
Shaanxi	1.147	0.260	0.984	1.166	0.299
Gansu	3.356	1.796	1.287	2.607	6.028
Qinghai	0.842	0.346	0.842	1.000	0.291
Ningxia	1.053	0.624	1.053	1.000	0.657
Xinjiang	1.864	0.601	1.124	1.658	1.121
Mean	1.223	0.748	0.988	1.237	0.914

### 4.3 Analysis of influencing factors

#### 4.3.1 Variable description

Five explanatory variables were selected (Table 7): GDP (per capita Gross Domestic Product), MI (Management Input ratio), RSI (Research Staff Input ratio), ICS (Importance of Competitive Sports), and ITR (Importance of Technological R&D).

**Table 7:** Descriptive statistics of variables.

Variable	Observations	Mean	Median	Min	Max
GDP	155	73,871	62,900	31,336	190,313
MI	155	20.94	20.63	10.23	40.34
RSI	155	68.86	85.00	0.00	100.00
ICS	155	95.66	96.43	72.31	99.47
ITR	155	0.3081	0.1800	<0.001	5.3300

#### 4.3.2 Regression results

The Tobit regression results are presented in Table 8.

**Table 8:** Tobit regression results.

Explanatory variables	Technical efficiency (Z^p^)	Pure technical efficiency (Z^p^)	Scale efficiency (Z^p^)
GDP	−1.95 ^(0.051)^	2.735 ^(0.006**)^	−2.983 ^(0.003**)^
MI	1.053 ^(0.292)^	−0.489 ^(0.625)^	1.623 ^(0.105)^
RSI	−3.629 ^(<0.001**)^	−1.545 ^(0.122)^	−4.369 ^(<0.001 ***)^
ICS	0.063 ^(0.950)^	−0.692 ^(0.489)^	−0.118 ^(0.906)^
ITR	−1.889 ^(0.059)^	−1.109 ^(0.267)^	−2.238 ^(0.025 *)^
Intercept	10.676 ^(<0.001***)^	23.445 ^(<0.001***)^	11.713 ^(<0.001***)^

***, **, and * denote statistical significance at the 1%, 5%, and 10% levels, respectively. Coefficients are reported to three decimal places; *p*-values are to three decimals, with those less than 0.001 marked as <0.001.*.

The Tobit regression results (Table 8) indicate that regional economic development (GDP) exerts a dual effect on the efficiency of S&T investment in competitive sports. GDP has a significant positive effect on pure technical efficiency (PTE) (coeff. = 2.735, *p* = 0.006), suggesting that more developed regions are better at managing and utilizing technological resources. Conversely, it has a significant negative effect on scale efficiency (SE) (coeff. = −2.983, *p* = 0.003), which may reflect diseconomies of scale or diminishing returns as the economic scale expands. Meanwhile, research staff input (RSI) shows a strong negative association with both technical efficiency (TE) (coeff. = −3.629, *p* < 0.001) and scale efficiency (SE) (coeff. = −4.369, *p* < 0.001), indicating that simply increasing research personnel does not translate into efficiency gains. Furthermore, greater emphasis on technological R&D (ITR) is also negatively associated with scale efficiency (coeff. = −2.238, *p* = 0.025). In contrast, the effects of management input (MI) and the importance of competitive sports (ICS) on the various efficiency measures did not reach statistical significance in this model.

#### 4.3.3 Robustness checks

Robustness tests, including substituting the dependent variable with Comprehensive Efficiency and performing bootstrap procedures (2,000 iterations), confirmed the stability of the key regression results. The direction and significance of the main predictors remained consistent.

## 5 Discussion

This study, grounded in the dual theoretical framework of the Resource-Based View (RBV) and Knowledge Conversion Theory, systematically examines the efficiency of provincial S&T investment in China's competitive sports from 2018 to 2022. The observed pattern of generally low efficiency alongside significant regional disparities cannot be attributed merely to the scale of investment; rather, it must be understood through the lens of resource conversion capability. While RBV emphasizes the heterogeneity and inimitability of strategic resources, this research reveals that possessing abundant S&T resources—such as funding, institutions, and personnel—is only a necessary condition. The critical factor lies in whether regional systems possess the organizational capability to effectively configure and activate these resources. For instance, provinces like Inner Mongolia and Jiangxi consistently remained on the efficiency frontier in certain years, suggesting they have developed unique pathways for embedding S&T elements into their local training ecosystems. Conversely, the fluctuations and periodic declines in efficiency observed in some economically developed provinces likely reflect an imbalance between resource expansion and growing management complexity. This points to a dual dilemma of “resource redundancy” and “diminished management effectiveness”. This finding deepens the traditional resource-based view by suggesting that the value of resources lies not only in their static endowment but, more critically, in the dynamic process of “resource orchestration”.

The significant negative correlation between research staff input (RSI) and efficiency warrants particular in-depth analysis. This finding might seem counterintuitive from a mere input-output perspective but finds a coherent explanation through Knowledge Conversion Theory. This theory conceptualizes knowledge creation as a four-stage process: socialization, externalization, combination, and internalization. The current sports science system appears to suffer from a disconnect, particularly in the stages of “combination” (systematizing explicit knowledge) and “internalization” (embedding knowledge into the tacit capabilities of individuals and organizations). An increase in research personnel, if not supported by effective knowledge conversion mechanisms—such as institutionalized dialogue between researchers and practitioners, collaborative research focused on practical competitive problems, and tailored knowledge dissemination pathways for coaches and athletes—can result in an “island effect” in knowledge production. A substantial volume of research outputs remains confined to papers and reports, failing to transform into practical solutions that enhance athletic performance or optimize training processes. This directly leads to the paradox of “increased input without corresponding efficiency gains”. Therefore, this result does not negate the value of researchers; rather, it sharply highlights a core deficiency in the current S&T support system: the weak linkage between research and training.

From a methodological standpoint, this study's integration of the DEA, Malmquist index, and Tobit models provides a workable framework for evaluating the efficiency of public S&T investment in sports, which is characterized by multiple inputs and outputs. While any model has its boundary conditions—for instance, the DEA assumption of homogeneous decision-making units is challenged by the vast socio-economic and sports-cultural disparities among Chinese provinces—this study partially mitigates the bias of attributing all heterogeneity to managerial inefficiency by introducing environmental variables like regional economic level and management structure into the second-stage Tobit regression. Subsequent research could employ methods like “meta-frontier analysis” to decompose efficiency sources more precisely while acknowledging technological differences between groups. Furthermore, the lagged nature of competitive sports outcomes means that current outputs may carry the effects of historical investments, posing a challenge for precise attribution. Future studies utilizing longer panel datasets and dynamic models could more clearly delineate the time-lag structure and long-term impact between S&T investment and athletic achievement.

Based on the above analysis and findings, this study carries clear policy implications. The primary direction is to shift the management logic of S&T investment from a “scale-oriented” approach to one focused on “efficiency” and “precise fit”. At the macro level, it is advisable to establish a differentiated resource allocation mechanism based on efficiency evaluation outcomes, guiding resources toward regions or domains with stronger configuration capabilities, and to set up cross-regional platforms for knowledge sharing and technology transfer to break down resource barriers. At the micro operational level, it is essential to construct an institutionalized ecosystem for “research-training integration”. This could involve creating dedicated roles such as “S&T Director” at key training bases, mandating that research projects be jointly proposed and implemented by research institutions and training teams, and establishing regular S&T service stations for sports teams. These measures would actively bridge the channel for knowledge to flow from the laboratory to the training ground. Finally, a national-level monitoring and evaluation system for the effectiveness of S&T in competitive sports should be established. Incorporating static efficiency scores and dynamic indicators like Total Factor Productivity change into the regular assessment system for sports development would create a continuous improvement loop of “evaluation-feedback-optimization”. This would ultimately drive the fundamental transformation of China's S&T support system for competitive sports from extensive growth to intensive, quality-driven development.

## 6 Strength and limitations

This study has several strengths. Methodologically, it integrates the DEA, Malmquist index, and Tobit models, enabling a comprehensive assessment that combines static efficiency measurement, dynamic productivity change analysis, and the identification of key influencing factors. Empirically, it employs extensive and authoritative panel data (2018–2022) across all 31 Chinese provinces, providing a solid foundation for a systematic national evaluation. Theoretically, applying the Resource-Based View and Knowledge Conversion Theory offers a coherent framework for interpreting the complex mechanisms behind efficiency disparities, moving the analysis beyond mere description to diagnostic insight.

The study also has several limitations. The findings, particularly those from the Tobit regression, indicate correlations rather than established causal relationships. The DEA model's assumption of homogeneous decision-making units simplifies the substantial contextual heterogeneity among provinces, even though a two-stage approach was used to mitigate this. Furthermore, inherent time-lags exist between S&T investment and measurable sports outputs (e.g., world champions), meaning the analysis captures the efficiency of the current support system rather than fully isolating the long-term causal impact of investment. Data constraints, including reliance on aggregated statistical yearbooks and the potential for measurement error, also pose limitations. Future research should employ methods such as meta-frontier DEA, distributed lag models, and mixed-methods case studies to better address heterogeneity, temporal dynamics, and causal mechanisms.

## 7 Conclusion

This study demonstrates that the efficiency of S&T investment in China's competitive sports system depends less on resource endowment than on regional capabilities for strategic resource orchestration and conversion into athletic performance. Grounded in the Resource-Based View and Knowledge Conversion Theory, our DEA-Malmquist-Tobit analysis of provincial panel data (2018–2022) reveals persistently low static efficiency (mean = 0.40) with pronounced regional disparities, while dynamic total factor productivity exhibits modest improvements offset by periodic volatility. Notably, research staff input demonstrates a significant negative association with efficiency, underscoring a systemic “knowledge translation gap” between scientific research and training practice. Regional economic development enhances pure technical efficiency but erodes scale efficiency, exposing inherent tensions between resource availability and managerial complexity. These findings collectively advocate for a policy paradigm shift from quantitative investment expansion toward optimizing strategic resource orchestration, contextual tailoring, and practical application of S&T resources to cultivate a more effective and sustainable ecosystem for competitive sports development in China.

## Data Availability

The original contributions presented in the study are included in the article/Supplementary Material, further inquiries can be directed to the corresponding author.
